# Four new markers of early thrombotic molecules and thromboelastography in prognostic evaluation of AMI patients undergoing PCI

**DOI:** 10.1186/s43044-026-00728-5

**Published:** 2026-03-11

**Authors:** Weisha Sun, Yi Gao, Weiran Sun, Qinhan Zhang, Xi Zhao, Xuan Jing, Chonghua Hao

**Affiliations:** 1 Shanxi Medical University, Taiyuan, China; 2 Shanxi Provincial People’s Hospital, Taiyuan, China

**Keywords:** Acute myocardial infarction, Thromboelastography, Thrombotic molecules, Conventional coagulation tests, Thrombomodulin

## Abstract

**Background:**

Early and accurate diagnosis and prognostic evaluation of acute myocardial infarction (AMI) are essential to improve clinical outcomes. Conventional coagulation tests (CCTs) are routinely used but have limited sensitivity and specificity in capturing the complexity of thrombus formation and fibrinolysis.

**Main body:**

Recent research has introduced a range of emerging biomarkers—including thrombomodulin, thrombin–antithrombin complex, plasmin–α2-plasmin inhibitor complex, and tissue plasminogen activator inhibitor complex—that reflect endothelial injury, thrombin generation, and fibrinolytic activity. In parallel, thromboelastography (TEG) has gained attention as a whole-blood assay that offers a dynamic and comprehensive view of the coagulation cascade. These new tools provide valuable information beyond that available from traditional tests and have been explored for their diagnostic and prognostic utility in AMI patients undergoing percutaneous coronary intervention. The combined assessment of these biomarkers and TEG parameters enhances risk stratification by reflecting both vascular injury and coagulation dysfunction. Furthermore, TEG supports individualized antiplatelet therapy by continuously monitoring clot formation, strength, and dissolution.

**Conclusion:**

Based on the available evidence, we conclude that the biomarkers—TM, TAT, PIC, and t-PAIC—demonstrate potential clinical value in providing a more refined assessment of thrombus formation and fibrinolytic activity and TEG provides an important basis for optimizing antiplatelet therapy by dynamically monitoring the coagulation process in whole blood.

## Introduction

Acute myocardial infarction (AMI) is a common cardiovascular disease with high prevalence and high lethality. Global deaths from cardiovascular disease have increased from 12.4 million in 1990 to 19.8 million in 2022, reflecting the contribution of global population growth and aging as well as preventable metabolic, behavioral, and environmental risks [1]. Percutaneous Coronary Intervention (PCI) is currently one of the main diagnostic methods for the diagnosis and treatment of AMI. Its main purpose is to restore blood flow to the coronary arteries through interventional manipulation, thereby saving myocardial tissue, reducing myocardial necrosis, and influencing patient prognosis. Even with the use of potent antiplatelet agents and drug-eluting stents, the risk of major adverse cardiovascular event (MACE) after PCI still exists, which seriously affects the prognosis of patients, and there is a lack of clinically valid prognostic predictors [2]. With the increase of patients with coronary heart disease and the continuous popularization and application of interventional techniques, the prevention and treatment of post-interventional complications have become a major hotspot of concern in the cardiovascular field [3]. Therefore, accurate prediction of reperfusion outcomes is essential for reducing the risk of MACE and improving patient prognosis.

**Fig. 1:**
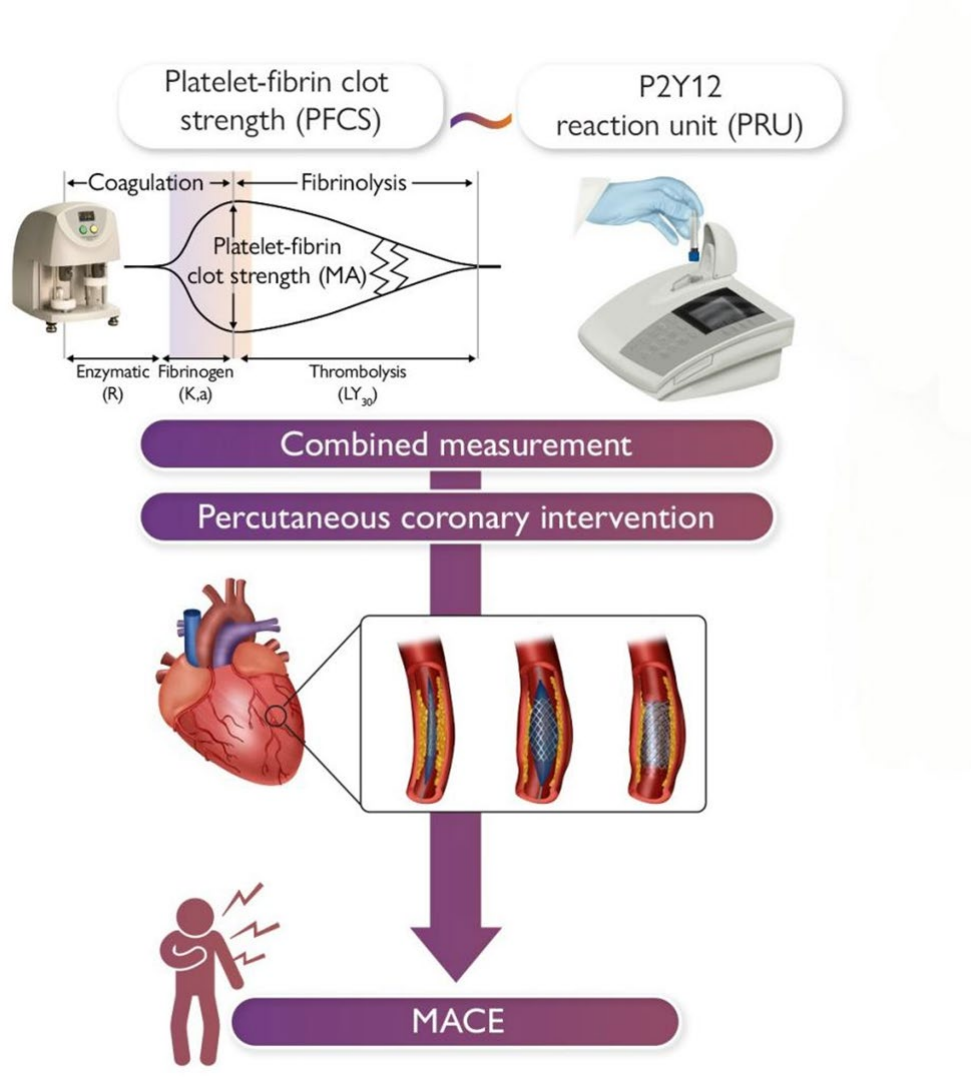
Combined measurement of platelet-fibrin clot strength (PFCS) and P2Y12 reaction units (PRUs) in predicting the risk of MACE after PCI. Reprinted with permission from Kwon et al., European Heart Journal 2024[34]. PCI: percutaneous coronary intervention; MACE: major adverse cardiovascular events.

Thromboelastography (TEG), a global, portable test for hemostasis [4], can be used in AMI for disease monitoring, treatment assessment, and prognosis prediction (Fig. 1). Several thrombo-fibrinolytic biomarkers—including thrombomodulin (TM), thrombin–antithrombin complex (TAT), plasmin–α2-plasmin inhibitor complex (PIC), and tissue plasminogen activator inhibitor complex (t-PAIC) [5]—has been increasingly studied for their role in reflecting the balance of coagulation and fibrinolysis during acute cardiovascular events. These indicators serve as important cofactors in the anticoagulation pathway with anti-inflammatory properties [6] and are able to provide a comprehensive picture of endothelial damage and the coagulation-fibrinolytic system [7].

## Purpose and methodology of the narrative synthesis

The aim of this review was to explore the published literature with a focus on analyzing the progress of the application of thrombotic biomarkers with TEG in the prognostic assessment of AMI patients undergoing PCI. A literature search was performed in PubMed, Chinese databases (https://creativecommons.org/licenses/by-nc-nd/4.0/), and Google Scholar databases for literature published between 1996 and 2024, including peer-reviewed articles and online preprints.

The following keywords and Boolean operators were used in various combinations: (“acute myocardial infarction” OR “AMI”) AND (“thrombomodulin” OR “TM” OR “thrombin-antithrombin complex” OR “TAT” OR “plasmin-α2 plasmin inhibitor complex” OR “PIC” OR “tissue plasminogen activator inhibitor complex” OR “t‑PAIC”) AND (“thromboelastography” OR “TEG”) AND (“prognosis” OR “risk assessment” OR “PCI”).

Inclusion criteria:

Original studies (clinical trials, observational studies, or cohort studies), reviews, or meta-analyses reporting on at least one of the four biomarkers (TM, TAT, PIC, t‑PAIC) in the context of AMI; Human studies; Publications in English or Chinese; Studies involving AMI patients undergoing PCI.

Exclusion criteria:

Animal experiments, basic science studies, or in vitro research; Studies not related to AMI; Case reports, editorials, or letters without original data; Duplicate reports or studies without full-text availability.

Eleven publications [23-27, 34, 36-38, 40, 52] provided in Table 1 describe the results of coagulation metrics studies comparing these biomarkers, TEG, and the conventional coagulation tests.

**Table 1:** Results of coagulation index studies in patients with AMI

Explore	Type of study	Overall results	Biomarkers Assessed	Sample Size
Lv et al. 2017 [23]	Cross-sectional study	Marked hypercoagulable state	TM, TAT, PIC, t-PAIC/Marked hypercoagulable	80 patients/intra-cardiac/reperfusion
Feng et al. 2024 [24]	Cross-sectional study	Hypercoagulability and Impaired fibrinolysis	TM, TAT, PIC, t-PAIC/ Hypercoagulability	368 patients/intra-cardiac/major adverse cardiovascular event
Wu et al. 2023 [25]	Cross-sectional study	Mild hypercoagulable state	TAT, PIC, TM, FRC, PLT/Mild hypercoagulable state	48 patients/intra-cardiac/major adverse cardiovascular event
Kosaki et al. 2018 [26]	Retrospective cohort study	Normal coagulation state	TM, TAT, PIC, t-PAIC, CCTs/Normocoagulable	400 patients/intra-cardiac/major adverse cardiovascular event
Carville et al. 1996 [27]	Prospective Cohort Study	Mild hypercoagulable state	TM, CCTs/Mild hypercoagulable	300 patients/intra-cardiac/Angina pectoris
Xu et al. 2019 [34]	Prospective Cohort Study	Hypercoagulable state	TEG, CCTs/Hypercoagulable	385 patients/intra-cardiac/major adverse cardiovascular event
Kwon et al. 2024 [36]	Prospective Cohort Study	Mild hypercoagulable state	TEG, CCTs/Mild hypercoagulable state	2512 patients/intra-cardiac/major adverse cardiovascular event
Yoshii et al. 2022 [37]	Prospective Observational Study	Mild hypercoagulable state	ROTEM, CCTs/Mild hypercoagulable	25 patients received blood transfusion therapy due to perioperative bleeding
Lee et al. 2023 [38]	Prospective Cohort Study	Hypercoagulability and Impaired fibrinolysis	TEG, CCTs/Hypercoagulability and Impaired fibrinolysis	2705 patients/intra-cardiac/major adverse cardiovascular event
Tu et al. 2023 [40]	Retrospective cohort study	Hypercoagulability	TEG, CCTs/Hypercoagulability	59 patients/intra-cardiac/major adverse cardiovascular event
Ng et al. 2024 [54]	Retrospective case-control study	Hypercoagulability	CCTs, CWA/Hypercoagulability	214 patients/intra-cardiac/Venous thromboembolism (VTE)

## Overview of AMI and MACE

### Disease definition and pathogenesis

AMI, a severe form of acute coronary syndrome (ACS), occurs when a coronary artery is partially or completely blocked by thrombus, with thrombin (Factor IIa) playing a key role. This leads to severe myocardial ischemia, injury, or necrosis [8]. AMI also triggers adverse cardiac remodeling, which can result in heart failure. Despite improvements in healthcare, AMI remains a leading cause of death worldwide [9]. Early and accurate diagnosis of chest pain, along with monitoring sensitive and specific markers, is crucial for effective treatment and prevention of unnecessary hospitalization [10].

MACE is defined as a composite endpoint of all-cause death, nonfatal myocardial infarction, ischemic stroke, and systemic embolism [11]. MACE often occurs after PCI and has an increased incidence of irreversible adverse events due to its mechanical injury, inflammatory cytokines, and other entry into the microcirculation.

### Coagulation status after the onset of AMI

AMI is primarily triggered by the rupture or erosion of atherosclerotic plaques, initiating platelet aggregation and activation of the coagulation cascade, which leads to thrombus formation and myocardial ischemia [12]. Less common mechanisms include coronary dissection, vasospasm, embolism, and microvascular dysfunction [13]. In the context of AMI, red thrombi respond well to rt-PA, while white and mixed thrombi, commonly seen in arterial circulation, often require combined antiplatelet and anticoagulant therapy due to their resistance to fibrinolysis [14].

While intensified antithrombotic therapy post-PCI reduces ischemic recurrence, it may increase bleeding risk [15]. In a multicenter randomized trial involving 2989 STEMI patients, Yan et al. [16] found no significant difference in ischemic or major bleeding events between the anticoagulation (PPA) and placebo groups (HR = 1.00), suggesting that routine PPA use is both safe and effective in low-to-moderate risk patients. This highlights the need for optimized anticoagulant selection combined with precise coagulation assessment to balance ischemic and bleeding risks. Therefore, accurate evaluation of a patient’s coagulation status is essential for balancing ischemic protection and hemorrhagic safety. Conventional biomarkers provide partial insight, but integrated approaches—such as thromboelastography (TEG) and novel thrombo-fibrinolytic markers—may offer more comprehensive and personalized risk assessment.

### Diagnostic criteria for AMI

The diagnostic criteria for AMI include a rise and fall in myocardial injury markers (cTnI, cTnT, or CK-MB) and at least one of the following: ischemic symptoms, ST-segment changes, pathological Q waves, or imaging evidence of myocardial damage or wall motion abnormalities [17]. Electrocardiogram (ECG) and cardiac biomarkers, especially cardiac troponins (cTn), are essential for diagnosis [18]. History, physical examination, and risk factor assessment also play critical roles [8]. Accurate AMI type identification is vital for treatment and prognosis [19]. Type 1 AMI, linked to plaque rupture, accounts for 60%-80% of ACS, while AMI without coronary obstruction requires different treatment [20]. Using ECG, biomarkers, and supplementary tests (e.g., coronary imaging and echocardiography), clinicians can better identify AMI types, assess damage, and guide treatment [21].

## Clinical application of thrombo-fibrinolytic biomarkers in acute myocardial infarction

### Physiological and pathological roles of TM, TAT, PIC, and t‑PAIC in AMI

Recent studies have demonstrated that TM, TAT, PIC, and t-PAIC—has shown clinical relevance in the early identification [22], prognosis evaluation, and risk stratification of AMI patients. Their physiological significance in reflecting the dynamic balance between thrombosis and fibrinolysis underlies their clinical utility, as summarized in Table 2.

**Table 2:** Overview of coagulation and fibrinolysis biomarkers assessed in AMI prognosis

Sports event	frame of reference	Primary Pathway	clinical significance
Thrombomodulin, TM	**3.8–13.3 TU/mL**	**Endothelial Injury**	**Markers of endothelial injury are used to assess bleeding and endothelial repair during treatment**,** and to evaluate therapeutic efficacy.** 1. **Regulation of vascular coagulation**: TM-thrombin complex 2. **Molecular marker of endothelial cell damage**: The level of TM is positively correlated with the extent of endothelial injury. 3. **Associated with systemic vascular disorders**: Elevated TM levels are observed in conditions such as renal disease, cancer, and coronary artery disease.
Thrombin-antithrombin complex, TAT	**< 4.0ng/mL**	**Thrombin Generation**	**Molecular markers of coagulation system activation indicate prothrombin activation and the initiation of thrombosis.** 1. **Early diagnosis of DIC (Disseminated Intravascular Coagulation)**: TAT levels are significantly elevated in 90% of DIC cases; normal TAT levels can help TAT levels are significantly elevated in 90% of DIC cases; normal TAT levels can help exclude DIC. 2. **Assisting in DIC classification and disease status assessment**: TAT helps in guiding timely treatment choices. 3. **Monitoring the effect of anticoagulation/antifibrinolytic therapy and reassessing thrombolytic treatment.** 4. **Thrombotic predisposition in conditions such as atrial fibrillation**,** and mitral stenosis combined with atrial fibrillation**: TAT is elevated in TAT is elevated in these thrombotic states. 5. **Thrombotic diseases (deep vein thrombosis**,** pulmonary embolism)**,** acute leukemia**,** and certain malignant tumors (e.g.**,** lung cancer)**: TAT levels can be significantly increased.
Plasmin-α2 plasmin inhibitor complex, PIC	**< 0.8ug/mL**	**Fibrinolysis**	**Early markers of fibrinolytic system activation**,** indicate the initiation of fibrinolysis and the formation of thrombosis**,** with TAT levels also likely elevated.** 1. **In DIC**: Secondary fibrinolysis is enhanced, leading to a significant increase in PIC. In primary fibrinolysis, PIC is elevated, but TAT levels do not increase. In primary fibrinolysis, PIC is elevated, but TAT levels do not increase. 2. **Monitoring thrombolytic therapy**: (e.g., t-PA, urokinase, streptokinase). 3. **Cancer (especially metastatic tumors)** can induce DIC, and acute promyelocytic leukemia is also prone to trigger DIC, leading to elevated PIC levels. 4. **Rheumatic diseases and systemic lupus erythematosus** can lead to increased PIC levels.
Tissue plasminogen activator inhibitor complex, t-PAIC	**Healthy males: <17 ng/mL; Healthy females: <10.5 ng/mL.**	**Fibrinolysis Inhibition**	**Comprehensive markers reflect both fibrinolytic system activation and endothelial cell injury.** 1. **Early diagnosis of DIC and VTE**: Compared to PIC, which reflects the final stages, t-PAIC can early detect the activation of the fibrinolytic system. 2. **Molecular marker of endothelial cell damage**: It can be used during treatment to assess the degree of endothelial repair. 3. **Monitoring the effectiveness of antifibrinolytic/anticoagulant therapy.**

### Clinical application of TM, TAT, PIC, and t‑PAIC in AMI

Building upon their physiological roles, these biomarkers have been increasingly explored for their prognostic implications in AMI.

#### Fibrinolytic activity – PIC and t-PAIC

Previous studies have demonstrated that markers of fibrinolytic activity are also dysregulated in AMI. Lv et al. [23] found that PIC and t-PAIC levels were significantly elevated in AMI patients compared to healthy controls, suggesting active fibrinolysis. This elevation preceded detectable changes in conventional coagulation tests, indicating their potential for early diagnosis.

Moreover, t‑PAIC’s elevation is associated with fibrinolytic inhibition, which may predispose to persistent thrombosis despite endogenous fibrinolysis. Its clinical relevance lies in identifying patients at risk of impaired thrombus resolution. A recent study of 368 AMI patients found that elevated plasma t-PAIC levels were significantly associated with higher mortality and increased risk of MACE over 6 months [24]. With a cut-off value of 15.3 ng/mL, t-PAIC showed strong predictive ability for mortality (AUC 0.871) and moderate prediction for MACE (AUC 0.671). These findings highlight t-PAIC’s value as a prognostic biomarker in AMI.

#### Coagulation activation – TAT

TAT levels are elevated during thrombin activation, and several studies link TAT to worse outcomes in AMI. Wu et al. [25] observed that TAT, when combined with TM, had high predictive accuracy for MACE (AUC: 0.884), indicating its utility in risk stratification.

#### Endothelial injury – TM

Kosaki et al. [26] further reported that TM levels were independently associated with major adverse cardiovascular events (MACE) in post-PCI patients, even after adjusting for cardiac and renal function. Carville et al. [27] found that plasma TM concentrations in AMI patients were 4–20 times higher than in controls. In patients presenting early with chest pain, significantly elevated TM levels (*p* < 0.01) were observed, supporting TM’s value in early detection of endothelial damage and active thrombosis. These findings underscore the prognostic value of TM beyond traditional risk markers.

#### Combined marker utility

While each marker provides mechanistic specificity, studies have shown that their combined detection improves sensitivity and specificity for early AMI diagnosis and risk assessment. This supports a panel-based approach to biomarker evaluation, particularly in emergency settings.

In summary, these biomarkers, when interpreted in light of their distinct pathophysiological roles, offer valuable tools for early detection, risk stratification, and prognostic evaluation in AMI. Future work should further explore their synergistic diagnostic potential and develop standardized algorithms for clinical integration.

## TEG in acute myocardial infarction

### TEG principles

**Fig. 2:**
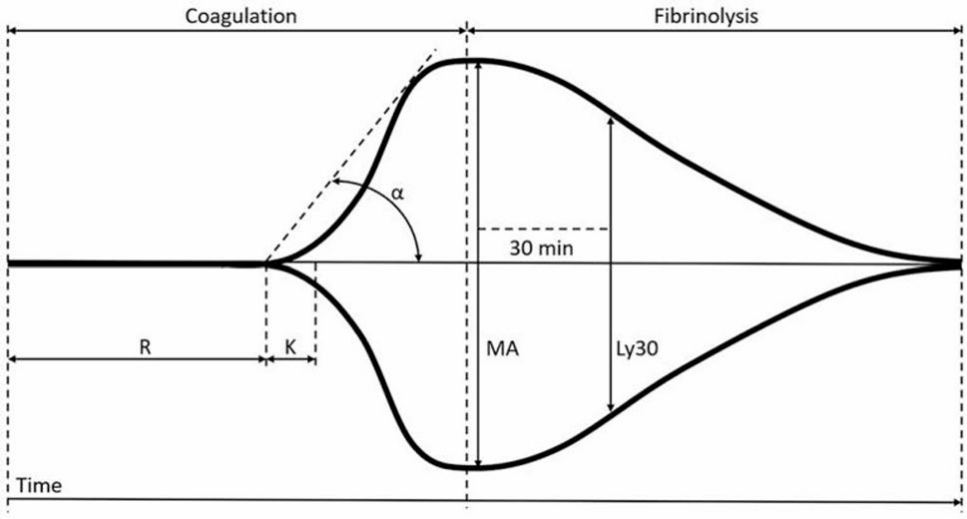
Schematic representation of thrombelastography (TEG) parameters. R time, the time from placing the sample in the TEG analyzer to the initial fibrin formation; K time, the interval from the initial clot formation to the fixed hardness level, set at 20 mm; α angle, the angle between the horizontal line at the center of the TEG trace and the line tangent to the 20 mm mark; Ly30, the percentage decrease in clot size 30 min after thrombus formation; Maximum amplitude (MA), The maximum width reached by the trace, reflecting the maximum strength and stability of the clot

TEG is a laboratory technique for testing the coagulation function of whole blood that allows dynamic monitoring of the entire process from the initiation of coagulation to fibrin formation, thrombus stabilization, and fibrinolysis [28]. The main parameters include (Fig. 2): R time: reflects the initiation of coagulation, prolongation suggests anticoagulation or hypocoagulability. K time and alpha angle: reflect the rate and intensity of thrombus generation. Maximum Amplitude (MA): indicates the contribution of platelets to thrombus strength.Ly30: reflects the fibrinolytic process and is a key indicator for assessing thrombus stability. TEG is able to provide a real-time, comprehensive assessment of blood coagulation and fibrinolysis by simulating in vivo blood flow conditions [29]. As well as by measuring the rotational motion produced when a blood sample coagulates, it assesses the role of blood coagulation factors, fibrinogen, platelets, etc., on the coagulation response, as well as the effect of anticoagulant and fibrinolytic activity [30]. Not only can it detect abnormalities in coagulation function, but it can also help physicians provide important references in treatment decisions for various diseases in the clinic, especially in the fields of surgery, acute and critical care medicine, hematology, liver disease, and cardiovascular disease to assist in the development of more rational treatment plans [31−32].

### Comprehensive monitoring of coagulation status

In major cardiovascular events such as AMI, timely and accurate assessment of patient’s coagulation status is crucial for the development of effective clinical treatment strategies, and TEG has shown its important clinical application value in this process. Studies have shown that TEG can reflect the coagulation abnormality of AMI patients with high sensitivity, help clinics to analyze its causes, and take targeted preventive and therapeutic measures, which may effectively reduce the mortality and recurrence rate of patients [33]. In particular, using TEG to monitor the effect of antiplatelet therapy before and after operations such as PCI can effectively reduce the occurrence of MACE and improve the efficiency and safety of treatment [34−35].

**Fig. 3:**
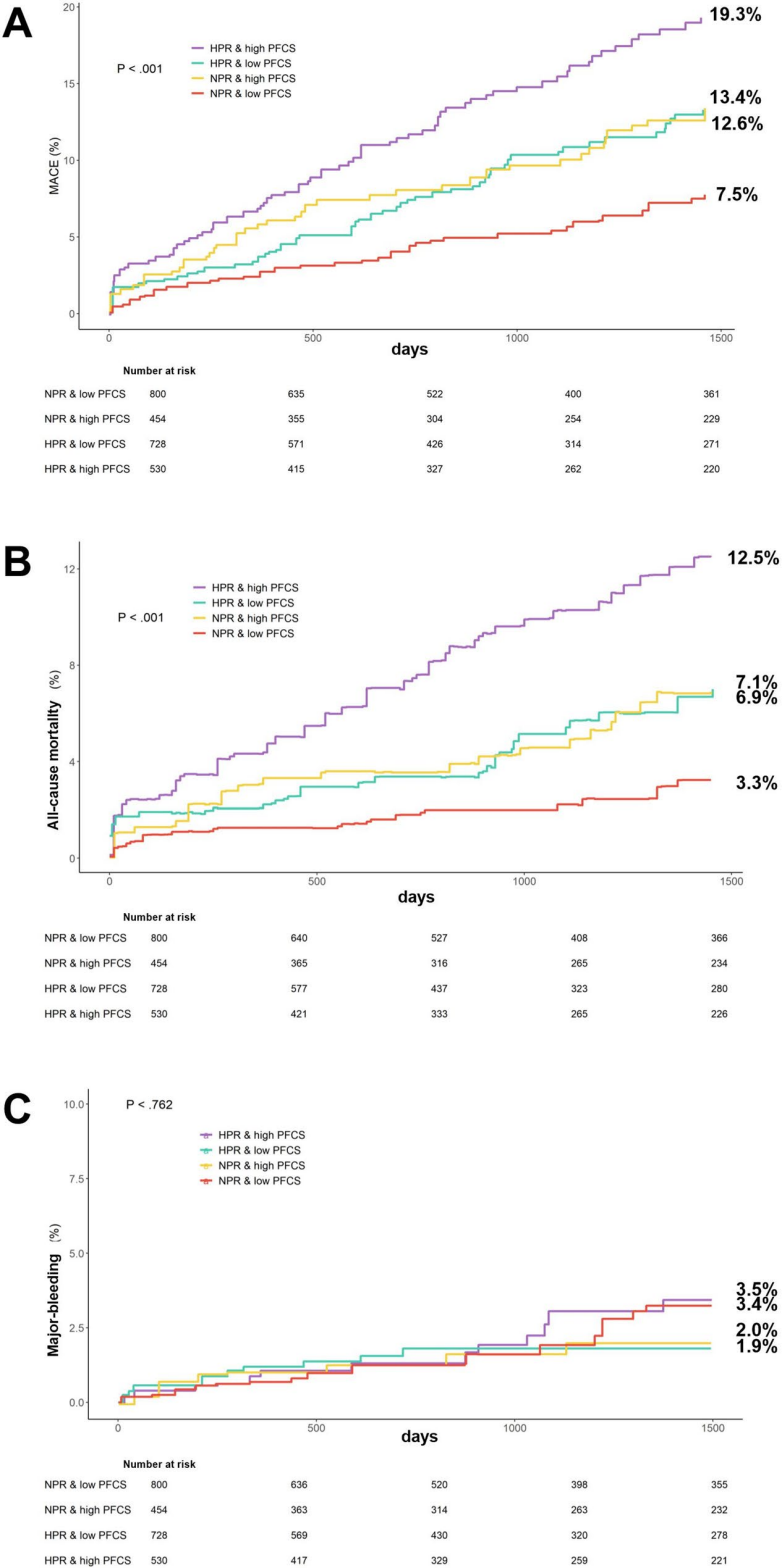
Survival curve analysis after PCI in 4 groups of AMI patients. **A** major adverse cardiovascular events; **B** all-cause mortality; **C** major bleeding

In a retrospective cohort study in 2024, Osung et al. [36] enrolled 2512 patients with AMI and used TEG to measure platelet-fibrin clot strength and VerifyNow to measure platelet reactivity, with up to 4 years of follow-up for MACE and hemorrhage outcomes. The study found that platelet reactivity and clot strength showed a superimposed effect in predicting acute ischemic events. The risk of MACE was significantly higher in the high platelet reactivity group and the high platelet-fibrin clot intensity group compared with the normal platelet-fibrin clot intensity group. The normal platelet-fibrin clot strength group was associated with a higher risk of major bleeding compared with the normal phenotype in the high platelet reactivity group and the high platelet-fibrin clot strength group (HRadj 3.12; 95% CI 1.30–7.69; *P* = 0.01) (Fig. 3). Thus after PCI, personalized antithrombotic therapy based on platelet reactivity and clot strength may improve long-term prognosis.

TEG has gained widespread use as a validated assessment tool in thrombus monitoring and therapy, especially in clinical settings such as cardiac surgery where precise management of coagulation is required, and its role is becoming more prominent. Yoshii et al. [37] evaluated the correlation between thromboelastometry parameters and CCTs in a prospective observational study of 25 patients who required cardiac surgery. The results of the study showed that the correlation between clot stiffness and CCTs was highly significant (*r* = 0.91, 0.93, and 0.93; *p* < 0.001, respectively). In addition, plasma factor XIII also showed a strong correlation with thromboelastometry results (*r* = 0.66 and 0.73; *p* < 0.001, respectively). The investigators further suggest that the use of thromboelastometry parameters in the transfusion management of cardiac surgery has been repeatedly shown to significantly reduce the transfusion requirements of patients undergoing extracorporeal circulation and may serve as an important tool in guiding transfusion decision-making in the clinical setting.

**Fig. 4:**
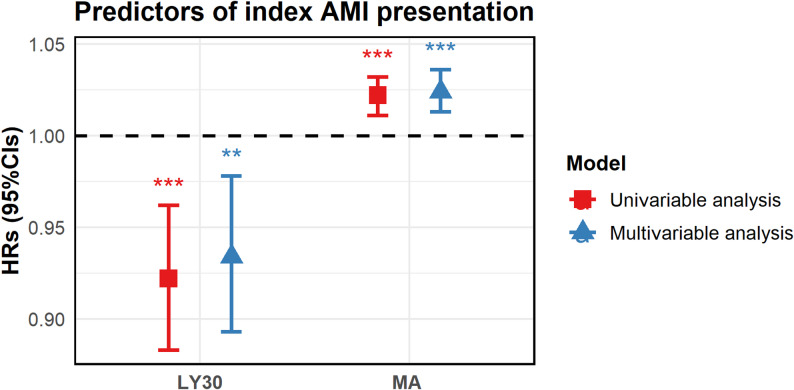
Predictors of index AMI performance. Multivariable analysis revealed that MA (per 1 mm increase: OR, 1.024; 95% CI 1.013–1.036; *P* < 0.001) and LY30 (per 1% increase: OR, 0.934; 95% CI, 0.893–0.978; *P* = 0.004) were independently associated with MACE in AMI patients. The model showed a c-statistics of 0.69

To further investigate whether hypercoagulable state may be associated with AMI, Lee et al. [38] evaluated MACE for up to 4 years from consecutive patients undergoing PCI, grouped according to disease severity (AMI vs. non-AMI), with thrombotic index measured by TEG, and showed that compared to non-AMI patients, the AMI patients had higher platelet-fibrin clot strength and lower fibrinolytic activity in AMI patients. The occurrence of index AMI events was associated with MA and LY30 (as shown in Table 3): MA: 1.024-fold (OR) risk of morbidity per 1-mm increase, 95% confidence interval (CI) 1.013–1.036; *P* < 0.001; LY30: 0.934-fold (OR) risk of morbidity per 1% increase, 95% confidence interval (CI) 0.893–0.978; *P* = 0.004 (Fig. 4).

**Table 3:** Predictors of TEG results in patients with AMI

	Univariate analysis		Multivariate analysis	
	Ratio (95% CI)	*P*-value	Ratio (95% CI)	*P*-value
MA (per 1 mm increase)	1.022 (1.011–1.032)	< 0.001	1.024 (1.013–1.036)	< 0.001
LY30 (per 1% increase)	0.922 (0.883–0.962)	< 0.001	0.934 (0.893–0.978)	0.004

Data are expressed as number of events (%). The c-statistics of multivariable model was 0.69. AMI, acute myocardial infarction; CI, confidence interval; LY30, percentage of the clot that has lysed 30 min after the time of maximum amplitude; MA, maximum amplitude; Multivariable logistic regression model was constructed using variables with *P* < 0.1 in univariable analyses

**Fig. 5:**
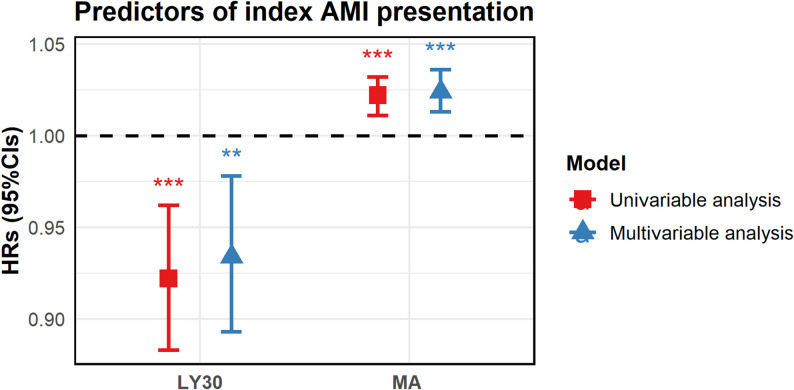
Comparison of 4-year MACE according to TEG parameters. Comparison of cumulative incidence and Kaplan–Meier curves of MACE at 4 years according to **A** hypercoagulability and **B** impaired fibrinolytic activity

The presence of high platelet-fibrin clot strength (MA ≥ 68 mm) and low fibrinolytic activity (LY30 < 0.2%) were synergistically associated with the development of MACE (Fig. 5). Furthermore, in multivariate analysis, the combined phenotype of “MA≥68 mm” and “LY30 < 0.2%” suggested that enhanced thrombogenicity was a major predictor of MACE after PCI in the AMI group.

**Fig. 6:**
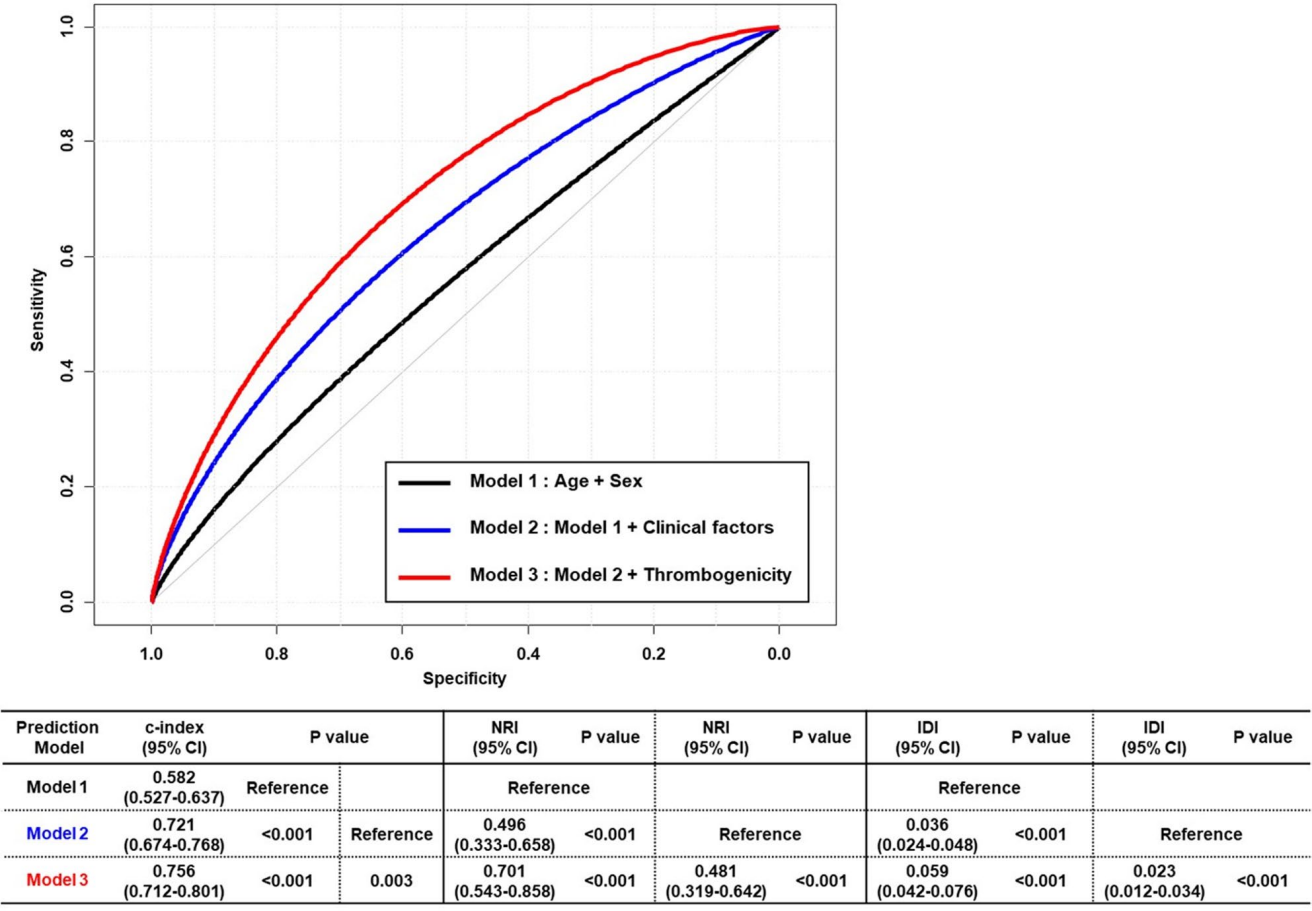
Incremental Prognostic Value of Thrombogenicity for MACE Risk at 4 Years. Prognostic values of models predicting 4-year MACE were compared using Harrell’s c-index, NRI, and IDI. Model 1 included the clinical variables of age and sex. There was significant increase in discrimination and reclassification ability with addition of other clinical variables of hypertension, diabetes mellitus, dyslipidemia, current smoker, chronic kidney disease, previous PCI, previous stroke, high sensitivity C-reactive protein level, potent P2Y12 inhibitor, beta blocker, angiotensin blocker, and statin (model 2). Model 3 with thrombogenicity (MA ≥ 68 mm and LY30 < 0.2%) showed further increase in discrimination and reclassification ability for 4-year MACE. The incremental prognostic value of model 3 was consistent when compared with model 2. Abbreviations: CI = confidence interval; LY30 = percentage of the clot that has lysed 30 min after the time of maximum amplitude; MA = maximum amplitude; MACE = major adverse cardiac event; NRI = net reclassification index; IDI = integrated discrimination index; PCI = percutaneous coronary intervention

The incremental prognostic value of thrombogenicity, as indicated by the combined TEG parameters (MA ≥ 68 mm and LY30 < 0.2%), was compared with a clinical variable model in a separate analysis (Fig. 6). The final model, incorporating TEG parameters, showed a significant improvement in both discrimination and reclassification ability, with a c-index of 0.756 (*P* < 0.001), Net Reclassification Improvement (NRI) of 0.701 (*P* < 0.001), and Integrated Discrimination Improvement (IDI) of 0.059 (*P* < 0.001). Similarly, a previous study [36] reported that integrating platelet-fibrin clot strength (PFCS, defined as MA > 68 mm) with NPR or HPR status—both derived from VerifyNow testing and closely related to MA (NPR typically corresponds to moderate platelet inhibition with MA values in the normal range of 50–70 mm, while HPR is often associated with MA > 70 mm or > 65 mm in some studies)—significantly enhanced the predictive performance of the model, further underscoring the additive value of thrombogenicity markers.

These results underscore the superior prognostic performance of thrombogenicity as assessed by TEG compared to conventional clinical variables. This illustrates the potential of TEG to improve risk stratification and clinical decision-making in AMI, especially when used alongside other biomarkers and clinical models.

### Guidance on treatment

TEG has shown potential application value in the treatment of AMI, as it can assess blood coagulation status and the resistance of platelets to medication, helping to reduce the occurrence of MACE and improving treatment safety and efficacy [39]. Xu Jingjing et al. [34] conducted TEG testing on 6,785 PCI patients and found a dual drug resistance rate of 5.67%, which was associated with factors such as gender, elevated lipids, and inflammation. Tu Jingjing et al. [40] conducted a retrospective study on 118 AMI patients and found that the monitoring group had higher R and K times than the control group, but shorter α angle, MA, and LY30. The MACE incidence in the monitoring group was 1.69%, significantly lower than the control group’s 8.47%. The study indicates that TEG can effectively assess PCI treatment outcomes, help determine thrombosis risk, and reduce the occurrence of MACE. By monitoring coagulation status, TEG can detect coagulation dysfunction early and guide personalized anticoagulant therapy, thereby improving the treatment outcomes for critically ill patients such as those with AMI [41−42].

In the management of patients with cardiovascular diseases such as AMI, TEG can not only be used as a portable auxiliary diagnostic tool but also as an important reference for treatment and prognostic assessment, reflecting some specific changes in disease progression, thus providing complementary information for clinicians [43]. By providing comprehensive coagulation function information, it offers more precise treatment guidance and prognostic assessment for clinical practice [44].

Beyond diagnosis, TEG has been used as a practical bedside tool for therapy guidance and prognostic assessment in cardiovascular patients [41–43]. Although conventional risk scores such as GRACE and TIMI are widely adopted and recommended for prognostic assessment in AMI, they are based on static clinical variables and therefore cannot reflect real-time hemostatic dynamics, whereas TEG provides unique advantages by detecting states like hypercoagulability and fibrinolytic shutdown. TEG-derived parameters—especially maximum amplitude (MA) and fibrinolytic index (LY30)—have shown predictive value for ischemic and bleeding outcomes. For instance, MA ≥ 68 mm is frequently used to define hypercoagulability, while LY30 < 0.2% indicates fibrinolytic impairment [38].

Despite these advantages, TEG remains underutilized in routine AMI care and is not yet integrated into formal risk stratification models. A major challenge is the lack of standardized threshold definitions across populations, as well as limited consensus on when and how to apply these tools. Current studies have identified a TEG maximum amplitude (MA ≥ 68 mm) as a marker of hypercoagulability, which has been correlated with increased thrombotic events—such as stent thrombosis—in high-risk subgroups, suggesting that TEG could be especially useful in guiding escalation or de-escalation of antithrombotic therapy in these patients [45]. These findings suggest that TEG may be especially valuable in tailoring antithrombotic strategies for high-risk AMI patients, rather than being applied universally in routine care.

To bridge this gap, future work should focus on defining validated cutoffs, improving test accessibility, and developing prospective algorithms that incorporate TEG and thrombotic biomarkers into decision-making pathways. Notably, Spadafora et al. [46] showed that in-hospital bleeding significantly impacts post-discharge therapy and long-term prognosis in ACS, highlighting the urgent need for dynamic tools like TEG to help clinicians balance ischemic and bleeding risks in a more nuanced and patient-specific manner. In summary, integrating TEG into clinical workflows based on well-defined thresholds and targeted use in high-risk patients could improve individualized therapy and long-term outcomes in AMI management.

## Conventional coagulation tests in acute myocardial infarction

### The assessment items of conventional coagulation tests

Conventional coagulation tests (CCTs) include Prothrombin Time (PT), Activated Partial Thromboplastin Time (APTT), Fibrinogen (Fib), and Thrombin Time (TT). PT reflects the activation of the extrinsic coagulation pathway and is evaluated through the International Normalized Ratio (INR), which is crucial for assessing the effects of vitamin K antagonists [47]. APTT assesses the activation of the intrinsic coagulation pathway. Fib reflects the supply status of coagulation materials. TT is used to evaluate the conversion of fibrinogen to fibrin and to monitor the presence of anticoagulants in plasma [47]. CCTs play an essential role in the diagnosis and treatment of AMI by assessing coagulation function and guiding anticoagulant therapy.

### Strengths and limitations

In terms of advantages, the detection technology of CCTs is well-established and easy to perform, providing rapid coagulation status information for clinical use, which is of great significance in guiding anticoagulant therapy and other treatments [48]. In addition, through these basic tests, it is possible to initially understand whether coagulation function exists in patients, which can provide a basis for further diagnosis and treatment.

However, CCTs also have certain limitations. First, they are affected by multiple factors and have relatively low specificity and sensitivity, which may result in some AMI patients with coagulation abnormalities not being detected in time [48]. Secondly, CCTs are unable to differentiate between hypocoagulation and hypercoagulation and cannot adequately reflect the immediate and complex changes in hemostasis, and therefore may lead to overtreatment [49]. It has also been shown that CCTs only assess the first 5% to 10% of fibrin formation and do not assess clot strength/stability, natural anticoagulant activity, or the complex interactions between endothelium, platelets, and coagulation factors [50] − [51]. For specific clinical situations, such as assessing the immediate effects of anticoagulation therapy, CCTs are not as sensitive and accurate as some of the novel coagulation assays [52].

Despite the aforementioned limitations, CCTs remain essential in diagnosing and managing AMI. These tests help assess bleeding and embolic risks, guiding clinical treatment. During PCI, CCTs like Activated Coagulation Time (ACT) and APTT are used to monitor anticoagulation levels, typically within a therapeutic window of 50–75 s (1.5–2.5 times the upper normal limit) [53]. Chen et al. [54] found that elevated clot waveform analysis (CWA) parameters in AMI patients correlated with adverse outcomes, suggesting their potential as prognostic markers. Baskaran et al. [55] showed that higher preoperative inflammatory markers were associated with greater morbidity and mortality, highlighting the role of inflammation in MACE. Therefore, correct interpretation of these markers is crucial for optimizing treatment and improving prognosis. As medical technology advances, the integration of new thrombotic markers and thromboelastography is expected to improve AMI diagnosis and management accuracy.

### Comparison of sensitivity and specificity

CCTs are widely used in screening for bleeding disorders, prethrombotic state examination, diagnosis of diffuse intravascular coagulation (DIC) and prognostic estimation of anticoagulation therapy [56]. In contrast, TEG and the four new markers of early thrombotic molecules are novel indices that have gained increasing attention in the diagnosis and management of coagulation disorders in recent years. Studies have shown that CCTs can only assess procoagulant abnormalities, while they are unable to evaluate anticoagulant abnormalities. In contrast, TEG markers demonstrate significantly higher sensitivity than conventional coagulation tests, providing a new pathway for earlier prediction and diagnosis of coagulation dysfunction in certain scenarios [56]. The specificity of TEG lies in the fact that it can provide a more comprehensive and accurate coagulation function than the conventional CCTs through the comprehensive assessment of thrombus formation, strength, stability, and lysis process. Comprehensive and accurate coagulation function than conventional CCTs. Unlike CCTs, which mainly evaluate a single factor in the coagulation process, TEG is able to comprehensively analyze all aspects of coagulation, platelet aggregation, clot formation, and lysis, and is particularly valuable in identifying various coagulation states. This enables TEG to effectively assess not only thrombus formation but also bleeding risk, thus providing more guidance for clinical decision making [57].

The biomarker panel demonstrates superior sensitivity in detecting subtle coagulation abnormalities, especially in the early stages of AMI and other cardiovascular diseases, and provide early warning of possible thrombosis. Their specificity lies in their ability to accurately reflect a patient’s coagulation status by assessing specific coagulation factors in the blood. For example, TM, as an endothelial damage marker, can directly reflect the degree of damage to the vascular endothelium and thus infer the potential risk of thrombosis. Since TM is highly correlated with thrombosis-related changes, it has high specificity and can help physicians determine the risk of thrombosis more accurately [58].

TAT is closely related to thrombosis, and its elevation indicates activation of thrombin in the blood. This indicator is effective in differentiating between different types of coagulation abnormalities and helps physicians to clarify the presence of persistent thrombosis in patients, especially in patients with AMI, where an elevated TAT often implies a higher risk of thrombosis. Because TAT reflects thrombin activity, it is highly specific [59].

Therefore, from the perspectives of sensitivity and specificity, both these novel biomarkers and CCTs have their unique advantages and limitations in the diagnosis and treatment of AMI, which need to be considered comprehensively on a case-by-case basis in clinical practice.

## Summary and outlook

In conclusion, plasma hypercoagulability from platelet activation and fibrinolysis system damage is central to cardiovascular diseases. TEG and the four new thrombotic markers help assess these abnormalities and detect subtle coagulation changes early, including MACE after PCI in AMI patients. Both play a vital role in clinical guidance, being widely used, easy to perform, accurate, and enabling real-time coagulation monitoring. Compared to conventional tests, they offer higher sensitivity and specificity.

Despite the promising potential of novel biomarkers in improving the accuracy of coagulation assessments, they come with certain limitations. For instance, biomarkers such as TM and TAT are highly specific but may not be sufficiently sensitive for detecting early-stage coagulation abnormalities in some patients. Furthermore, the clinical implementation of these biomarkers remains limited by cost, complexity, and the need for standardized protocols to ensure consistency across different laboratories and clinical settings. Similarly, while TEG offers a dynamic and comprehensive evaluation of coagulation, its clinical utility is also affected by several constraints. These include inter-operator variability, dependence on technical expertise, and limited availability in routine clinical practice. The integration of these tools into widespread clinical use will require overcoming these challenges and ensuring that healthcare professionals are adequately trained to interpret the results accurately. The integration of these biomarkers into routine clinical practice will require overcoming these challenges, as well as ensuring that healthcare professionals are adequately trained to interpret the results.

The integration of these biomarkers into routine clinical practice will require more robust clinical evidence demonstrating their added value over traditional coagulation tests. Multi-center trials focusing on diverse patient populations and specific clinical outcomes, such as long-term prognosis and MACE rates, will be crucial. Standardizing testing methods and reducing costs will also be necessary to make these tests more accessible. Moreover, clear guidelines for their clinical use will need to be developed, ensuring that these biomarkers are used appropriately in different stages of disease management, from diagnosis to prognosis and treatment monitoring.

As medical technology advances, the adoption of these novel biomarkers, along with thromboelastography, has the potential to significantly improve the diagnosis and management of AMI, offering more precise and individualized treatment strategies. In the near future, we envision a shift toward a more personalized medicine approach, where these biomarkers, combined with patient-specific data and AI-driven models, could facilitate early intervention, guide more accurate risk stratification, and optimize therapeutic outcomes. Continued research and innovation will be pivotal in realizing this vision, ultimately leading to better patient outcomes and a reduction in the burden of cardiovascular diseases globally.

## Data Availability

No datasets were generated or analysed during the current study.

## Abbreviations

ACS: Acute Coronary Syndrome
AMI: Acute Myocardial Infarction
AUC: Area Under the Curve
c-index: Concordance Index
CI: Confidence Interval
CK-MB: Creatine Kinase-MB
CCTs: Conventional Coagulation Tests
cTnI: Cardiac Troponin I
ECG: Electrocardiogram
HPR: High Platelet Reactivity
HR: Hazard Ratio
IDI: Integrated Discrimination Improvement
K time: Clot Kinetics Time
LY30: Lysis at 30 min
MA: Maximum Amplitude
MACE: Major Adverse Cardiovascular Events
NRI: Net Reclassification Improvement
NPR: Normal Platelet Reactivity
OR: Odds Ratio
PCI: Percutaneous Coronary Intervention
PPA: Prophylactic Parenteral Anticoagulation
rt-PA: Recombinant Tissue-type Plasminogen Activator
STEMI: ST-Elevation Myocardial Infarction
TAT: Thrombin–Antithrombin Complex
TEG: Thromboelastography
TM: Thrombomodulin
t-PAIC: Tissue Plasminogen Activator Inhibitor Complex
